# Structural development and energy dissipation in simulated silicon apices

**DOI:** 10.3762/bjnano.4.106

**Published:** 2013-12-20

**Authors:** Samuel Paul Jarvis, Lev Kantorovich, Philip Moriarty

**Affiliations:** 1 School of Physics and Astronomy, University of Nottingham, Nottingham NG7 2RD, United Kingdom; 2 Department of Physics, King’s College London, The Strand, London WC2R 2LS, United Kingdom

**Keywords:** apex structure, atomic force microscopy, DFT, dissipation, hysteresis, NC-AFM, silicon, spectroscopy, tip structure

## Abstract

In this paper we examine the stability of silicon tip apices by using density functional theory (DFT) calculations. We find that some tip structures - modelled as small, simple clusters - show variations in stability during manipulation dependent on their orientation with respect to the sample surface. Moreover, we observe that unstable structures can be revealed by a characteristic hysteretic behaviour present in the *F*(*z*) curves that were calculated with DFT, which corresponds to a tip-induced dissipation of hundreds of millielectronvolts resulting from reversible structural deformations. Additionally, in order to model the structural evolution of the tip apex within a low temperature NC-AFM experiment, we simulated a repeated tip–surface indentation until the tip structure converged to a stable termination and the characteristic hysteretic behaviour was no longer observed. Our calculations suggest that varying just a single rotational degree of freedom can have as measurable an impact on the tip–surface interaction as a completely different tip structure.

## Introduction

The theoretical treatment of chemical interactions at the single atom level has driven considerable progress in NC-AFM over the past decade. Through understanding the interactions between the AFM tip and sample surface, the chemical interactions present in AFM images [1–5], manipulation experiments [6–10], and, more recently, submolecular investigations of planar molecules [11–12], have been revealed. In covalent systems in particular, density functional theory (DFT) calculations have been extremely successful in explaining the fundamental interactions that underpin NC-AFM experiments [2–3,13–16]. Moreover, atomistic simulations remain essential to many current studies in covalent [17–19] and ionic [20–21] systems because of the inherent difficulties in determining the tip apex structure from purely experimental evidence. In contrast, on metal surfaces the requirement to use atomistic simulations for tip identification is not always as critical. For instance, there has been significant recent progress in developing experimentally driven methods to determine or engineer the tip structure with the use of CO molecules either adsorbed to the scanning probe tip [11], or used to reverse image a metallic tip apex by using the so-called carbon oxide front atom identification method (COFI) [22]. Such techniques provide an intuitive way in which to analyse and prepare the scanning probe tip. Similarly, reverse imaging can be employed on semiconductor surfaces, such as Si(111)-7×7 [23–24]. A comparison with either the COFI method or DFT calculations, however, is usually required to obtain the same level of confidence.

Semiconductors with covalent bonds remain one of the most promising systems for the advancement of atom-by-atom manipulation strategies in multiple dimensions and at room temperature. This is evidenced by numerous studies, which have shown the manipulation of single atoms in both lateral and vertical directions, which was made possible by the strong covalent nature of the bonding [25]. As such, understanding the AFM tip structure and successfully modelling experimental observations remains critical to furthering this goal. Several methods have been used to successfully model complicated tip structures such as variations in tip structure [16,26], chemical species [17,27] and, more recently, the directional dependence of reactive tips [18,28].

The orientation of the tip is rarely considered in theoretical work because of the high computational cost of running multiple simulations, although some do exist [29–30]. Therefore results are generally only presented for tip structures at a single orientation, even though modifying the tip–surface alignment can also strongly affect calculated tip-force *F*(*z*) curves and the hysteresis pathways followed by the tip and surface structures [28]. For instance, the bulk-like rear structure of tip apices is almost always aligned parallel to the surface for convenience when designing the tip. There is no reason to expect, however, that the experimental tip apex will follow the same rules. Therefore there is a clear constraint on current theoretical simulations due to the huge number of possible orientations that *a single* tip apex can adopt relative to *any* surface, even surfaces with perfectly symmetric dangling bond protrusions, let alone due to variations in tip apices.

Energy dissipation in NC-AFM measurements has most effectively been explained by adhesion hysteresis due to deformations in the tip–sample junction originating from bistable defects [31–33] or by structural relaxations within the larger structure of the AFM tip [34–35]. Dissipation is measured if the positions of some of the atoms (either in the surface, tip, or both) on approach and retraction are different, with the same atoms returning to their original positions at the end of the oscillation cycle. Observations of large dissipation signals of the order of electronvolts have been attributed to chain formation on insulating surfaces [36] and significant structural rearrangements of *both* the tip and sample over each oscillation of the AFM tip [16,37]. It has also been shown that in some cases the dissipation may be apparent – an instrumental artefact caused by mechanical coupling between the sensor and the piezo actuator [38].

In the current study we use the Si(100)-c(4×2) surface as a prototypical system, chosen because of its known dissipative behaviour in NCAFM experiments [8,13,37,39]. In particular, we have previously shown that a large variety of tip types are possible on the Si(100) surface, each demonstrating a different tip–sample interaction, and importantly, each exhibiting markedly different levels of measured dissipation [40]. Here we examine the effect that simple rotations of the simulated cluster can have on the tip–sample forces and the long-term stability of the tip apex. We observe that the rotation of the simulated tip cluster around the surface normal axis can have a dramatic effect on the stability of the tip apex such that at particular alignments permanent structural deformations occur which lead to new, stabilised tip geometries. We find that a tip prone to this behaviour demonstrates enhanced hysteresis in calculated *F*(*z*) data, dependent *only* on deformations within the tip apex, until complex structural rearrangements move the geometry into a more stable state. This suggests that even when varying just a single rotational degree of freedom, the difference in tip–surface interactions can be as significant as for a completely different tip structure.

## Simulation details

Our investigation is performed with ab initio density functional theory (DFT) simulations carried out by using the SIESTA code [41], which uses a double-zeta polarized basis set in the generalized gradient approximation with a Perdew–Burke–Ernzerhof density functional and norm-conserving pseudopotentials. Due to the relatively large size of the unit cell only a single |**k**| = 0 point was used for sampling the Brillouin zone. The atomic structure was considered relaxed when forces on atoms fell below 0.01 eV/Å. To obtain calculated *F*(*z*) curves the silicon tip clusters were placed at an initial vertical position of 8 Å above the Si(100) surface upper dimer atom. The vertical distance, *z*, is defined as the distance between the surface upper dimer atom and the lowest atom of the tip structure *prior* to relaxation. To ensure a smooth evolution of the tip structure and to avoid missing any of the hysteresis pathways, the tip was moved in quasi-static steps of 0.1 Å towards the surface and then retracted in the same way. At each point the vertical forces acting on the fixed tip atoms were summed up to give the total force that acts on the tip.

## Results and Discussion

**Figure 1:**
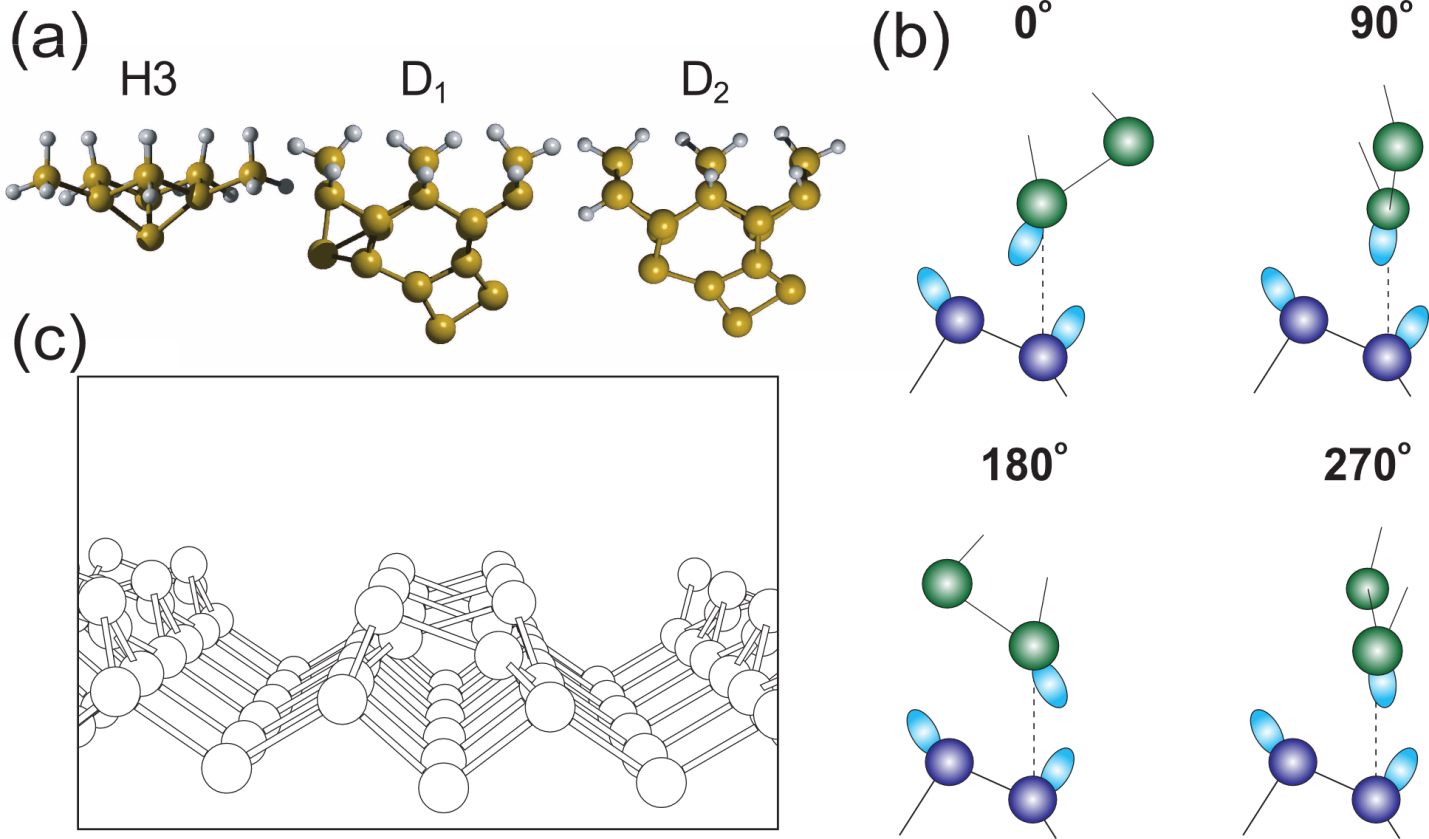
The three tip structures considered, a structurally rigid ‘H3’ termination, and two dimer-terminated tips, are shown in (a). D_1_ is relaxed with an additional stabilising atom as compared to D_2_. (b) *F*(*z*) was calculated for four rotations of the dimer tips with respect to the surface dimers. Note that due to the symmetry of the surface 90° and 270° are equivalent, but are still calculated independently for control. (c) A ball-and-stick model of the upper layers of the Si(100)-c(4×2) surface.

The structures considered in this study, and the characterisation process, are illustrated in Figure 1. The three tip structures considered, and a ball-and-stick model of the Si(100)-(c4×2) surface are shown in Figure 1a and Figure 1c. We consider three tip clusters that are commonly used to describe silicon tip apices, the so called “H3” structure and two dimerised silicon tip clusters. The dimerised tip in particular can be modified through inclusion of an atom on one side of the cluster which, as will be described below, has a stabilising effect on the tip. We are therefore able to model a high and low stability dimerised tip, which we label D_1_ and D_2_ respectively (see Figure 1a). It has previously been shown [28] that *F*(*z*) measurements can be used to characterise the tip structure through the examination of the energy dissipation during the dimer manipulation. A similar method is implemented in this work to assess the evolving structure of a silicon tip. In the current instance the tips are rotated through angles up to 360° around the surface normal axis, either positioned above the down, or up atom of a surface dimer. The angled nature of the Si(100) surface dangling bonds, particularly on the structurally rigid “up” dimer atom, allows us to easily investigate the effect of the tip-cluster alignment by rotations around a single axis, without having to consider the many other degrees of freedom available that would become more important on symmetric surfaces. The *F*(*z*) curves are calculated at four tip-surface alignments (see Figure 1b). This procedure is used not only as a theoretical assessment of tip stability, but also highlights that the rotational alignment of the tip relative to the surface, in some cases, can dramatically affect the chances of a major structural rearrangement.

### Energy dissipation in small apex clusters

**Figure 2:**
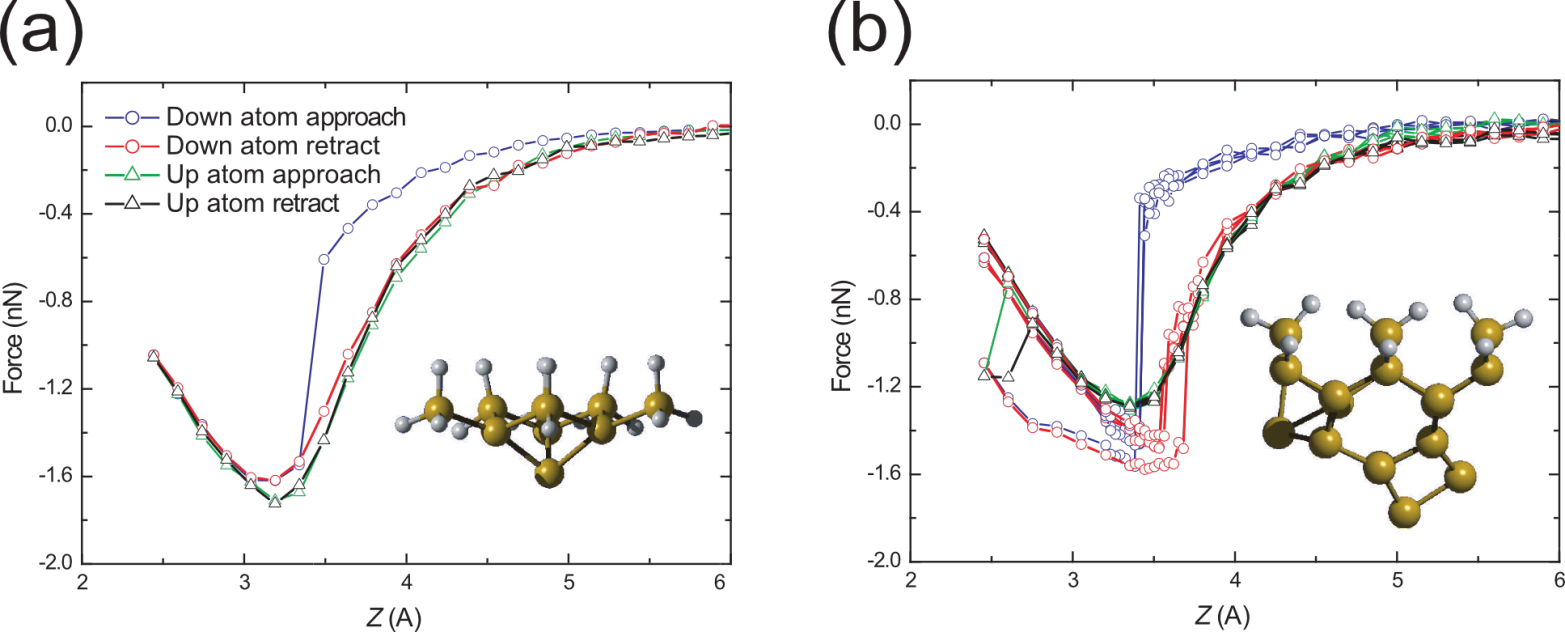
Simulated *F*(*z*) curves for the (a) H3 and (b) D_1_ tip structures taken above the up (green and black triangles) and down (red and blue circles) atoms of a surface Si(100) dimer. Curves in (b) of the same colour correspond to the different orientations of the tip with respect to the surface dimer as described in Figure 1b. It can be seen that the D_1_ tip shows little variation upon rotation around the surface normal axis. The H3 tip contains a symmetric apex and does not produce variation when rotated, therefore only a single rotation is shown.

Presented in Figure 2 are simulated *F*(*z*) curves taken with the H3 (a) and D_1_ (b) tips positioned above the up (green and black triangles) and down (red and blue circles) atoms of a surface Si(100) dimer. An in-depth description of the origins of the calculated force profile have been given elsewhere [8,13,42]. The key points, however, are summarised below. For tip apices positioned above the up dimer atom, a typical *F*(*z*) curve is observed with indistinguishable approach and retraction profiles (see, for example, 2a). When positioned above the down atom of the surface dimer, however, at a certain tip–sample distance a threshold force is met and a sharp jump is observed in the *F*(*z*) curve, which corresponds to a switching of the surface Si(100) dimer from a bond angle of approximately +19° to about −19°. For the remainder of the approach, and the subsequent retraction, the force profile follows that of the stable up dimer atom, a clear indicator of the successful switching event. Figure 2a depicts spectra that were taken with the high-stability H3 structure, which is used as our reference for a structurally rigid tip, which shows no variation upon rotation.

**Figure 3:**
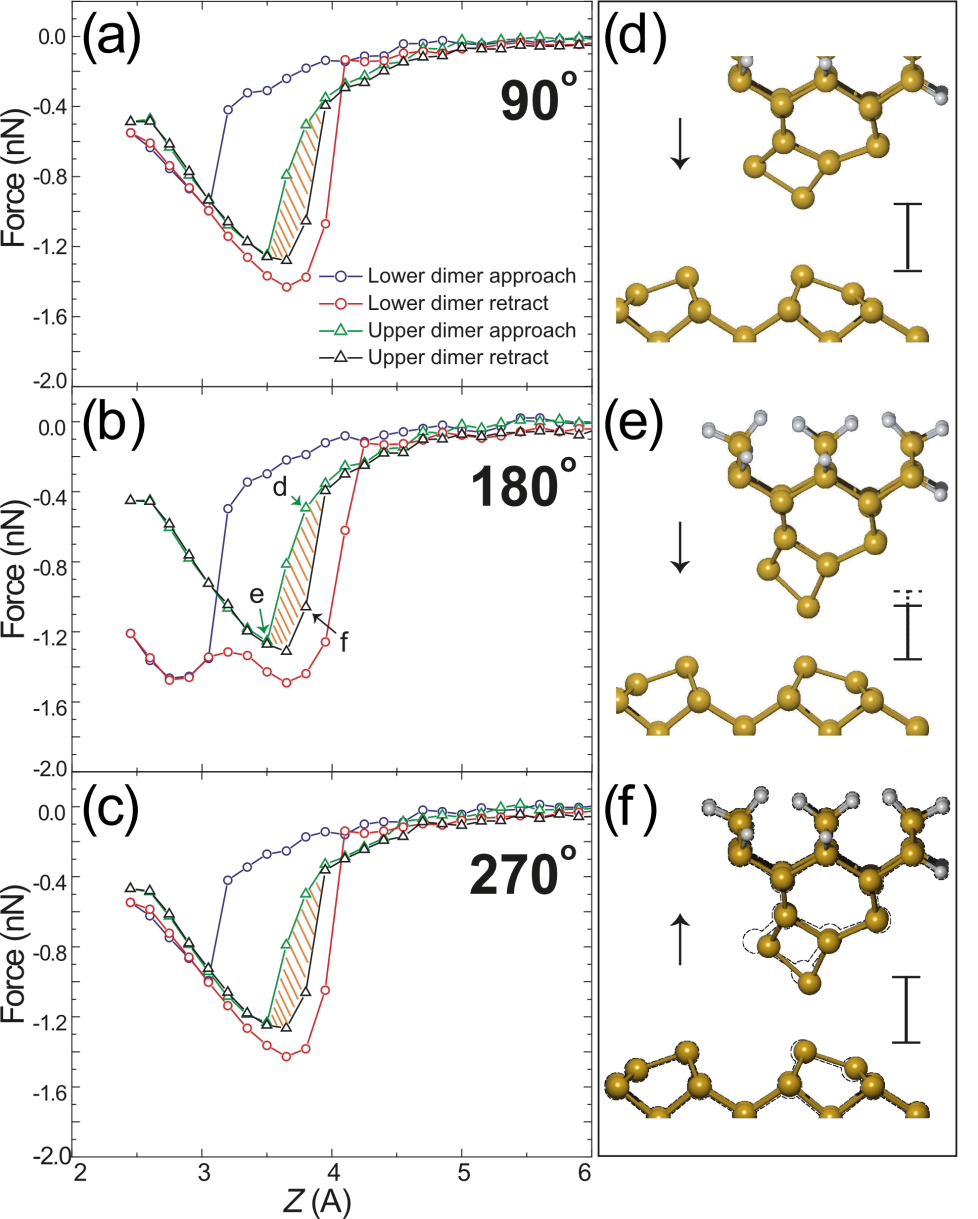
Simulated *F*(*z*) curves for the D_2_ tip at rotations (a) 90°, (b) 180°, and (c) 270°. The energy dissipation is significantly increased, and is critically also observed for the up atom site. Ball-and-stick snap shots are shown (d) within and (e) after the region of hysteresis as indicated in (b) during tip approach. (f) Ball-and-stick snap shot during retraction at the same position as (d), which is shown as a dashed outline, illustrating the alternative structural pathway taken by the tip, thus causing the observed hysteresis.

For the asymmetric D_1_ tip, even though the tip–surface alignment varies upon rotation around the surface normal axis, its structure is very stable and we observe minimal variation in the simulated *F*(*z*) curves. A small deviation is calculated only when the tip is rotated to the position we define as 180° (see Figure 1b), in which *both* of the atoms within the tip and surface dimers are able to interact with each other at very close approach. More interesting behaviour arises when we carry out the same simulations with the D_2_ apex as is shown in Figure 3. In this case a significant increase in energy dissipation (over a single cycle) is calculated for the down atom position of the tip (red and blue circles) amounting to an average 74% increase, from 0.39 eV to 0.68 eV relative to the more stable D_1_ cluster. The increase in hysteresis corresponds to hysteretic tip-deformations throughout the simulated *F*(*z*) curve. For the D_2_ tip, even though a significant level of dissipation is observed in the down atom position (a typical indicator of dimer manipulation [8,13]), the dimer, part way through the flipping process, in fact returns to its original state. This is noticeable as a sharp decrease in force during the retract curve. For successful manipulation, the target down atom of the dimer must be “pulled” high enough such that the up and down atoms trade places, switching the dimer buckling angle. The tip–dimer interaction for the D_2_ tip, therefore, is not sufficient to pull the down atom high enough to instigate manipulation [39,42–43].

Particularly interesting observations are made when the D_2_ tip is positioned above the structurally rigid up atom of the Si(100) dimer. Even though the surface atom remains mostly stationary throughout the approach–retraction calculation, a significant level of energy dissipation is calculated that amounts to 0.17 eV over a single cycle. The calculated *F*(*z*) curves taken above the up Si(100) dimer atom are shown in Figure 3 (approach: green triangles, retraction: black triangles). Ball-and-stick snap shots, at the positions marked in Figure 3b, are shown in (d–f) within and after the region of hysteresis. Although the surface dimer remains in the same position, it is clear that the D_2_ tip experiences significant deformation, which pulls the apex downwards into a narrower shape. The geometry shown in Figure 3f is taken at the same *z* position as (d), during retraction from the surface. From the calculated geometries we can see that the tip structures in (d) and (f) differ, thus modifying the tip–surface interaction, which in turn leads to the observed hysteresis. This theoretical result is very similar to experimental observations on the Si(100) surface that recorded a dissipation of up to 0.5 eV/cycle [40] for a tip that demonstrated a “dimer-tip”-type atomic resolution [44]. It has also been shown [34] that very large simulated tip clusters demonstrate the same behaviour, which is attributed to more permanent structural changes that are likely to occur within the much larger experimental tip. The difference we observe, therefore, is that no permanent structural change is required to observe a significant dissipation, even in much smaller silicon clusters.

This result has significant implications for understanding the origin of experimental observations of dissipation. Unlike the hysteresis observed for the down atom position (occurring over the single oscillation cycle when dimer manipulation takes place), *all* oscillation cycles, in which the point of closest approach falls below 3.5 Å will demonstrate hysteresis. Thus tip-dependent dissipation, even with very simple, small tip clusters such as the D_2_ tip, should be noticeable on *any* surface, which further confirms the assumption that the tip structure plays the dominant role in many experimental dissipation observations.

### Enhancing tip stability via surface indentation

Examination of the tip geometries in our simulations suggest that the increase in *F*(*z*) hysteresis is driven by significant structural rearrangements. Our calculations suggest that the D_2_ tip potential energy surface (PES) contains a number of shallow minima, which are separated by small barriers. Upon interaction with the surface the PES distorts in such a way that some of the barriers collapse, which opens a path for the tip to transform from one configuration to another. As a result the D_2_ tip provides alternative structural pathways during approach and retraction. Clusters that demonstrate a greater stability do not allow for the atomic rearrangements that are required for the additional hysteresis, because the barriers that separate the different minima on the PES of these clusters are not reduced sufficiently upon interaction with the surface. Therefore, in some instances, the presence of a tip-hysteresis may act as an identifier for a potentially unstable tip configurations.

Tip indentation is a commonly applied technique to improve the quality of tips in NC-AFM, and in turn to modify the quality of the image. The process typically involves gentle indentations of the tip by 1–2 Å into the surface relative to the Δ*f* feedback *z* position. As the tip is indented into the surface either material transfer, or atomic rearrangement can improve or worsen the quality of the AFM image. Thus far very few simulated studies have looked at the influence of surface indentation on the structure of the tip. Existing studies have either concentrated on coating the AFM tip with sample material [36] or sharpening very small and unstable silicon clusters [45]. Experiments that are carried out at room temperature are likely to have a sufficient energy available to heal any metastable tip states that might arise from such indentations. In this case simulated annealing [26] is usually sufficient for an accurate description. At low temperatures, however, where many exotic tip states have been observed [40], the available thermal energy becomes insufficient for restructuring the tip. Metastable tips are therefore far more likely to remain stable after a reconstruction of the tip.

**Figure 4:**
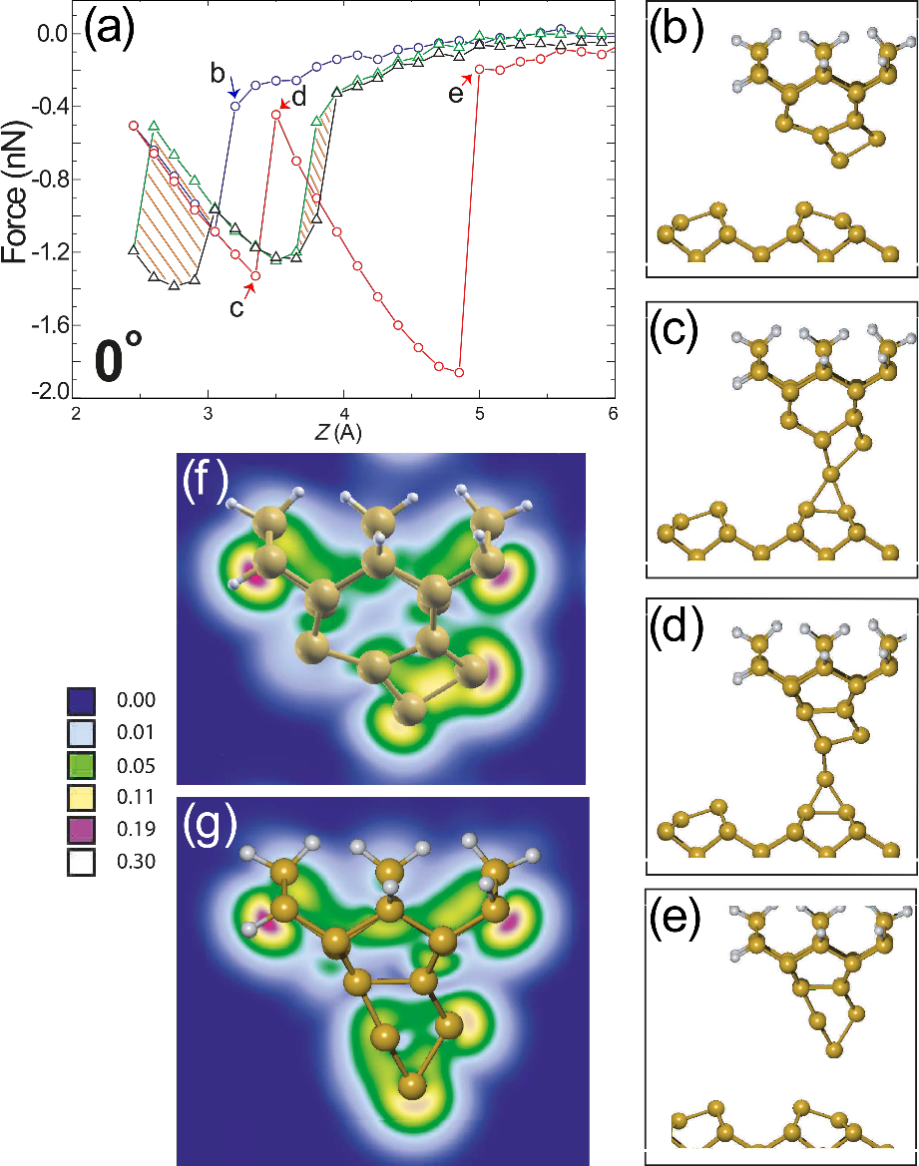
Structural development during tip indentation. (a) Calculated *F*(*z*) approach and retraction curves for the D_2_ tip at “0°” positioned above down (red and blue circles) and up (green and black triangles) surface dimer atoms. Calculation with the tip positioned above the down atom leads to structural rearrangement of the tip, noticed as discontinuities in the retract curve at ≈3.5 Å and ≈5 Å. The ball-and-stick model in (b) depicts the starting configuration of the tip during the approach, which is followed by the major stages in tip rearrangement during retraction (c–e). Partial electron density plots (calculated within the range 0–1 eV below the Fermi energy and plotted on a square root scale of electrons/Bohr^3^) of (f) initial and (g) final tip (D_2a_) configurations. Plots were made using the XCrySDen software [46].

In Figure 4 we show one such instance of tip development, in which the D_2_ tip, although stable for the simulations in Figure 3, undergoes major structural rearrangement when aligned at “0°”. The calculated *F*(*z*) curve at this position is shown in Figure 4a, in which two sharp jumps in force are present during retraction of the tip. Shown in (b–e) are geometries illustrating the major stages of tip rearrangement. Initially the tip configuration is as shown in (b), then the D_2_ tip forms a strong bond with the Si(100) surface dimer in (c), which results in similar deformations to those already shown in Figure 3. Upon retraction of the tip, however, the strong tip–surface bond (due to the favourable alignment with the surface [28]) introduces a significant strain to the tip structure, which develops it into a much sharper configuration relative to the initial D_2_ apex. Partial electron density maps, highlighting the dangling bond orbitals, are shown for the original D_2_ tip (f) and the sharpened structure (g) which we term D_2a_. A simple examination of the electron density plot reveals that the tip structure maintains a single prominent dangling bond orbital at its apex, which in principle should produce atomic resolution that is not significantly different from that to be expected from the initial tip structure. This may implicate that structural rearrangements of the tip may occur during the scan, which do not significantly affect the contrast and possibly remain largely unnoticed. We note that in previous studies the D_2_ tip remained stable during simulated spectroscopy [16,26], and in our own simulations, when positioned above the surface Si(100) up dimer atom, no structural changes are observed regardless of orientation. As such we believe that the D_2_ tip represents a plausible tip structure and a good candidate to test the orientation-dependent stability of the AFM tip cluster.

**Figure 5:**
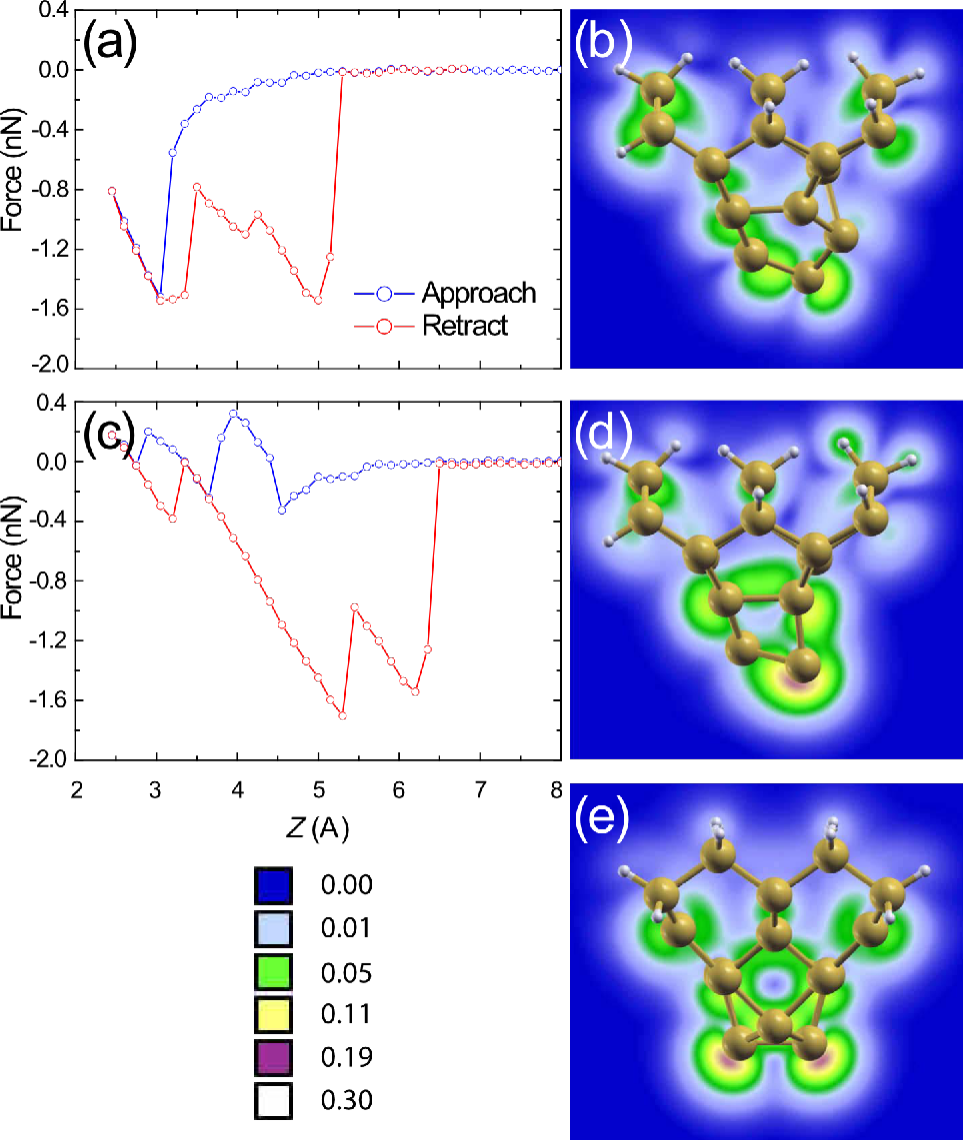
Continued development of tip D_2a_ via repeated tip indentations. (a) Calculated *F*(*z*) curve and (b) final tip configuration following indentation of the tip structure shown in Figure 4(g) leading to tip D_2b_. (c) Indentation of tip D_2b_ results in further modification noticeable as a series of sharp discontinuities in calculated *F*(*z*) prior to reaching a final, stable double tip shown from two perspectives in (d–e). Partial electron density plots shown with square root scale in units of electrons/Bohr^3^.

Experimentally, during Δ*f*(*z*) measurements or tip indentations carried out specifically to modify the apex, the scanning tip is constantly oscillating at a rate of a few kilohertz, often with an amplitude that is larger than the silicon interaction potential. Therefore, as the average *z* position is ramped towards the sample, the tip will undergo multiple cycles of approach and retraction. As a result, any structural development of the tip apex must occur over multiple approach–retraction cycles, until a stable configuration is obtained that no longer reconstructs. To properly reflect this process, DFT *F*(*z*) calculations were continued by using the D_2a_ tip without any modification of the system. Upon continuation we observe two further stages of structural development until a final stable configuration is reached. We term these two tips D_2b_ and D_2c_ and show the respective *F*(*z*) curves leading to their development in Figure 5.

For the transition from D_2a_ to D_2b_ shown in Figure 5a, a significant number of atomic rearrangements occur, visible as rapid variations in the retraction curve. In fact, the tip not only undergoes significant rearrangement, but actually deposits an atom onto the Si(100) surface. Material deposition is commonly observed during experimental imaging and spectroscopy, sometimes leading to improvements in image resolution, or often leading to instabilities and deterioration of image quality. The partial electron density plot in (b) illustrates the apex dangling bond structure of tip D_2b_, which appears to protrude at a large angle relative to the surface normal. This structure would likely lead to a complicated tip-surface interaction [40].

To test the stability of the D_2b_ tip a further calculation was carried out, just as for the D_2a_ structure, over the same deposited silicon atom. In this case the tip remained in the D_2b_ configuration without any further reordering. Assuming that this tip must now be stable when imaging the clean Si(100) surface, a final indentation was calculated above a clean Si(100) dimer. In this new position a further rearrangement of the tip was observed into a final, stable, configuration resulting in the *F*(*z*) curve that is shown in Figure 5c. For the D_2b_ to D_2c_ transition, extreme features are observed both in the approach and retraction sections of the calculated *F*(*z*) because of the complicated interaction between the tip and the surface Si(100) dimers. These features originate from the blunt structure of the tip interacting with two dimers on the surface during rearrangement. The D_2c_ tip structure is shown in Figure 5d and Figure 5e displayed from two perpendicular perspectives. This final tip configuration is found to be stable upon continued spectroscopy, which suggests that the tip apex is fully structurally developed. Interestingly, we find that the stable tip terminates in a dimer like structure, with each terminating atom located at very similar *z* positions. Each “dimer” atom is associated with a dangling bond protruding in the −*z* direction, angled away from one another as shown in Figure 5e. The cluster appears to be more crystalline than its predecessors, which may perhaps explain the dimer termination because of the (100) orientation of the base structure. It is interesting to note that a dimer-terminated tip such as this might be able to produce double-lobed surface features, doubling effects, or even fail to produce a well separated, understandable signal altogether. Such observations would depend on the surface under study, and on the separation of the surface atoms, which can be a particularly challenging problem when obtaining atomic resolution.

The simulated results in this paper provide interesting insights into the atomic rearrangements that take place during well known, and commonly observed, experimental processes. We examine the role that alternative structural pathways play during spectroscopy measurements, which might lead to tip-dominated dissipation observations, similar to previous suggestions [34]. Critically, however, our observations are made by using the small, simple tip clusters that are tractable using a DFT treatment of the system, rather than the larger, more complicated, structures that must exist experimentally. Therefore, if dissipation can be observed for clusters of this size, it is very reasonable to expect that the same processes can occur in much larger, and hence more realistic systems. This suggests that the tip structure could play a dominant role in many experimental observations of dissipation.

We also show that tip apices that demonstrate hysteretic behaviour may be inherently unstable during *F*(*z*) measurements, or soft tip indentations that lead to a major structural redevelopment of the tip apex. In our specific example, we show that a tip that may appear to be structurally stable at certain orientations with respect to the surface, might interact completely differently at another position. We suggest, therefore, that the examination of the tip orientation may be just as valuable as testing entirely new structures when making experimental comparisons. We expect that these results might apply not only for a rotation around the *z* axis (as studied here) but also around the *x* and *y* axes, which are not considered in this study. We also propose a method for developing tip structures, similar to experimental approaches, through repeated soft indentation into the surface until alternative stable structures are obtained. Such an approach might be particularly useful to build up a library of theoretical tip structures, which could assist the interpretation of experimental observations [40].
